# Protective Efficacy of Baculovirus Dual Expression System Vaccine Expressing *Plasmodium falciparum* Circumsporozoite Protein

**DOI:** 10.1371/journal.pone.0070819

**Published:** 2013-08-12

**Authors:** Mitsuhiro Iyori, Hiroki Nakaya, Katsuya Inagaki, Sathit Pichyangkul, Daisuke S. Yamamoto, Masanori Kawasaki, Kyungtak Kwak, Masami Mizukoshi, Yoshihiro Goto, Hiroyuki Matsuoka, Makoto Matsumoto, Shigeto Yoshida

**Affiliations:** 1 Laboratory of Vaccinology and Applied Immunology, Kanazawa University School of Pharmacy, Kanazawa, Japan; 2 Microbiological Research Institute, Otsuka Pharmaceutical Co. Ltd., Tokushima, Japan; 3 Department of Immunology and Medicine, USAMC-AFRIMS, Bangkok, Thailand; 4 Division of Medical Zoology, Department of Infection and Immunity, Jichi Medical University, Tochigi, Japan; Agency for Science, Technology and Research - Singapore Immunology Network, Singapore

## Abstract

We have previously developed a new malaria vaccine delivery system based on the baculovirus dual expression system (BDES). In this system, expression of malaria antigens is driven by a dual promoter consisting of the baculovirus-derived polyhedrin and mammal-derived cytomegalovirus promoters. To test this system for its potential as a vaccine against human malaria parasites, we investigated immune responses against the newly developed BDES-based *Plasmodium falciparum* circumsporozoite protein vaccines (BDES-PfCSP) in mice and Rhesus monkeys. Immunization of mice with BDES-PfCSP induced Th1/Th2-mixed type immune responses with high PfCSP-specific antibody (Ab) titers, and provided significant protection against challenge from the bites of mosquitoes infected with a transgenic *P. berghei* line expressing PfCSP. Next, we evaluated the immunogenicity of the BDES-PfCSP vaccine in a rhesus monkey model. Immunization of BDES-PfCSP elicited high levels of anti-PfCSP Ab responses in individual monkeys. Moreover, the sera from the immunized monkeys remarkably blocked sporozoite invasion of HepG2 cells. Taken together with two animal models, our results indicate that this novel vaccine platform (BDES) has potential clinical application as a vaccine against malaria.

## Introduction

Malaria is one of the world's most devastating infectious diseases and is a major killer of children under five years old in Africa. The World Health Organization estimates that malaria transmission is present in 98 countries, and that the disease causes 216 million clinical cases each year and 655,000 deaths [1]. The most advanced malaria vaccine candidate, RTS,S/AS01, a *Plasmodium falciparum* circumsporozoite protein (PfCSP)-based vaccine containing the specific adjuvant AS01 was developed many years ago [2]. This vaccine is based on an N-terminal truncated form of PfCSP, which is fused to the hepatitis B surface antigen on the virus-like particle (VLP) platform [3], [4]. The hybrid malaria-hepatitis B VLP is available lyophilized and undergoes point-of-use reconstitution with GlaxoSmithKline's AS01 adjuvant [3], [5]. The first Phase III trial of RTS,S/AS01 in African children reported approximately 50% efficacy against clinical and severe malaria in children 5 to 17 months of age, but a recent study reported lower efficacy (around 30%) in infants 6 to 12 weeks of age, an outcome that clearly needs to be improved [6], [7]. Although improvement of vaccine efficacy is urgently needed, rapid advancements in this goal may not be so easy because the protective immune mechanism of the RTS,S/AS01 is not fully understood. Thus, new and more effective vaccine delivery systems are urgently needed for malaria vaccines.

Recently, interest in an insect-infecting baculovirus has focused on its potential use as a vaccine and gene therapy vector [8], [9]. Thanks to its biosafety, low cytotoxicity, non-replication in transduced mammalian cells and an absence of preexisting antibodies (Abs) against it in humans, baculovirus has emerged as a novel gene delivery vector [8], [9]. Because the virus can induce innate immune responses in host cells, it has been well characterized as an adjuvant-free vaccine platform [10], [11]. This well-developed vaccine system can deliver the antigens of interest by several unique methods; for example, (i) the desired antigen can be expressed by the vector within host cells in a manner similar to DNA vaccines; (ii) the desired antigen can be displayed on the surface of the baculovirus, or (iii) the desired antigen can be expressed and displayed by the vector [8], [12].

We have developed a “baculovirus dual expression system (BDES)”, which drives malaria antigen expression by a dual promoter that consists of both baculovirus-derived polyhedrin and mammal-derived cytomegalovirus (CMV) promoters [13]–[16]. This system is very effective as an all-stage malaria vaccine platform. For pre-erythrocytic-stage parasites, the rodent malaria sporozoite BDES vaccine (AcNPV-Dual-PbCSP) provided a 100% protection against infection in the *P. berghei*-infected BALB/c rodent malaria model [13]. In addition, baculovirus vectors displaying the *P. yoelii* blood-stage antigens, merozoite surface protein 1 (PyMSP1_19_) and apical membrane antigen 1 (PyAMA1) conferred complete protection against *P. yoelii* rodent malaria [14], [15]. For the mosquito transmission-stage, BDES was utilized for expression of the transmission-blocking antigens Pfs25 and Pvs25 in the vaccines against the sexual stages of the parasites, which induced strong transmission-blocking responses by challenge using a transgenic *P. berghei* line expressing Pfs25 and Pvs25, respectively [16], [17].

In the present study we constructed a new BDES for expression of *P. falciparum* CSP (BDES-PfCSP). BDES-PfCSP elicited protective immune responses against PfCSP-transgenic parasites in mice, as evaluated by the level of protection conferred against sporozoite challenge infections. To advance this system for clinical use, we investigated the immune responses to this new vaccine in rhesus monkeys. BDES-PfCSP provided high levels of anti-PfCSP Abs with strong inhibitory activities against sporozoite invasion into HepG2 cells. Our results show the potential of this novel vaccine platform for application as a vaccine against malaria.

## Materials and Methods

### Ethics Statement

All animal care and handling procedures were approved by the Animal Care and Use Committee of Kanazawa University (No. 22118–1) and the Guidelines for Animal Care and Use prepared by Jichi Medical University (No. 09193). The non-human primate study was conducted in compliance with the Animal Welfare Act and other federal statutes and regulations relating to animals and experiments involving animals and adhered to the principles stated in the Guide for the Care and Use of Laboratory Animals, NRC Publication, 1996 edition. Monkeys were housed individually in standard squeeze-type stainless steel cages at a minimum floor space of 4.4 square feet. Cages were cleaned daily and sanitized biweekly. Monkeys were fed complete, commercially prepared monkey chow twice daily and mixed fresh vegetables and fruits at least four times per week. Chlorinated water was provided ad libitum via automatic watering valves. Animals participated in the nonhuman primate enrichment program in accordance with AFRIMS's SOP. All monkeys were given 5–20 mg/kg ketamine hydrochloride intramuscularly for anesththesia before blood was collected or animals were vaccinated. Monkeys were not sacrificed. At the conclusion of the study, animals were released from the protocol and back to the colony. All efforts were made to minimize suffering in the animals.

### Recombinant viral vaccines

To construct pTriEX-Dual-PfCSP-Tcell, the gene encoding amino acids 19–373 of PfCSP (i.e., lacking the N-terminal signal peptide and C-terminal glycosylphosphatidylinositol anchor sequences) was PCR amplified from *P. falciparum* 3D7 genomic DNA, and the resulting fragment was inserted into *EcoRI/Xma I* cut pTriEX-Dual-PbCSP [13]. To construct the plasmids of pCAP-CO-PfCSP-full and pCAP-CO-PfCSP-205, DNA sequences encoding PfCSP were synthetized to optimize codon usage in mammalian cells. All PfCSP sequences were modified to introduce a point-mutation where alanine at position of 361 was substituted with glutamic acid; this incorporated an H-2K^k^-restricted CD8 encoded epitope peptide into the constructs. Generation of recombinant baculoviruses and purification of baculovirus particles were conducted as described previously [13], [14]. To generate the adenoviral vector Adeno-COE/1–373, CMV*ie* promoter sequences, followed immediately by the gene encoding the full-length PfCSP were subcloned into pAd/PL-DEST (Invitrogen, Carlsbad, CA); viral purification and viral particle (VP) titrations were carried out according to the manufacturer's protocol. All other viral-related methods used herein are described in Materials and Methods S1.

### Immunizations

Balb/c mice were immunized intramuscularly three times at 3-week intervals with 10^8^ plaque forming units (PFU) of BDES-PfCSP, AcNPV-Dual-PbCSP or wild-type AcNPV (AcNPV-WT). A different group of mice were immunized twice at 3-week intervals with 10^10^ VP of the Adeno-COE/1-373 vaccine.

Malaria naive rhesus monkeys were selected and randomized into 6 groups. Each group was immunized intramuscularly, subcutaneously, or intradermally three times at 4-week intervals with 3×10^9^ PFU of BDES-PfCSP or AcNPV-WT.

### PfCSP-Tc/Pb sporozoite challenge

*Anopheles stephensi* mosquitoes (SDA 500 strain) were infected with PfCSP-Tc/Pb (Materials and Methods S1, [18]) by allowing them to feed on parasite-infected mice (Fig. S1). The condition of the parasites (in terms of their infectivity) to recipient mice was checked by exflagellation tests before blood feeding commenced. The sporozoite-positive rate in the salivary glands of the mosquitoes used for the challenge infections was over 58%. Two weeks after the third immunization, the mice were challenged by blood feeding with parasite-infected mosquitoes. For the challenge infections (via natural biting), three to seven mosquitoes were allowed to feed on the abdomen of each mouse for 20 minutes. The salivary glands from all blood-engorged mosquitoes in the PBS control group were dissected to confirm the presence of sporozoites. Where necessary, mosquito feeds were repeated with fewer mosquitoes until three to seven infected mosquitoes had bitten all of the mice.

### Statistical analysis

The Mann-Whitney U test and the Kruskal-Wallis non-parametric analysis were used to compare Ab titer levels. The Dunnett two-tailed *t*-test and the two-tailed Fisher's exact probability test were used to determine statistical differences in the protective efficacy of BDES. In all other experiments, statistical differences between the experimental groups were analyzed using the Student's *t*-test; *p* values <0.05 were considered statistically significant. Statistical analyses were performed using SPSS Statistics (version 19, IBM) and Prism version 5 (GraphPad Software Inc.).

## Results

### Construction of a baculovirus dual expression system for PfCSP

**Figure 1:**
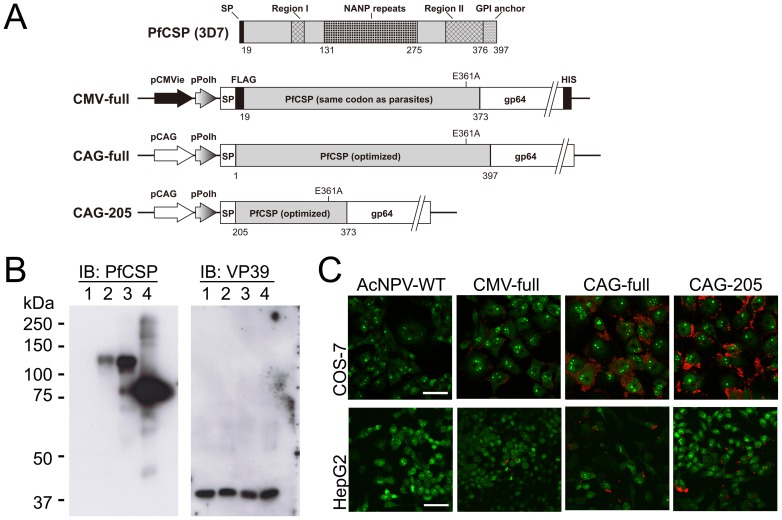
Schematic representation and expression analysis of BDES-PfCSP. (*A*) Expression of the PfCSP gene cassette was driven by the dual promoter, which consisted of the CMV immediate early enhancer-promoter (pCMV), or the CAG promoter (pCAG) as well as the polyhedrin promoter (pPolh). Both full-length *pfcsp* and the N-terminal truncated *pfcsp* were fused to the N terminus of the AcNPV major envelope protein *gp64* gene (gp64_21–512_). All BDES constructs also had the endogenous *gp64* gene in their genomes. SP, the gp64 signal sequence; FLAG, FLAG epitope tag; HIS, His epitope tag. (*B*) Western blot analysis of recombinant AcNPV particles. AcNPV-WT (lane 1), CMV-full (lane 2), CAG-full (lane 3) and CAG-205 (lane 4) particles were lysed, loaded onto an 8% gel and immunoblotted with the anti-VP39 Ab. The membrane was stripped and then reblotted with the anti-PfCSP mAb 2A10. (*C*) COS-7 cells and HepG2 cells were transduced with BDES-PfCSP at multiplicities of infection (MOI) of 500 and 100, respectively. After 48 hours incubation, such cells were stained with the 2A10 mAb conjugated to Alexa Fluor 594 (red). Nucleic acids in the cells were visualized by SYTO-13 (green). Bars, 50 µm.

Three types of BDES for heterologous expression of PfCSP genes (BDES-PfCSP) were constructed (Fig. 1A). The construct designated “CMV-full” harbored a gene cassette consisting of the gp64 major envelope protein signal sequence and the gene encoding PfCSP^19–373^ fused to the N-terminus of AcNPV gp64, which was driven by the CMV*ie*/polyhedrin dual promoter. The “CAG-full” vaccine construct was designed to express the codon-optimized PfCSP^1–397^ gene cassette under the control of the CAG/polyhedrin dual promoter. The “CAG-205” vaccine construct designed to express the codon-optimized PfCSP^205–373^ is an N-terminal truncated form of PfCSP, which is similar to the target sequence of the RTS,S vaccine [19].

### PfCSP expression in the BDES system

Western blotting showed that the anti-PfCSP mAb 2A10 recognized PfCSP expressed by CMV-full, CAG-full and CAG-205 constructs (Fig. 1B). The relative molecular mass of CMV-full was about 127 kDa, which is slightly bigger than that of CAG-full, whereas CAG-205 was around 85 kDa (Fig. 1B). Densitometry showed that the quantities of the PfCSP antigens displayed on CAG-full and CAG-205 were over 4-fold and 8-fold higher than that of CMV-full, respectively, when normalized against the viral protein VP39 using anti-VP39 polyclonal Abs (Fig. 1B).

To examine the ability of BDES vaccines to drive PfCSP expression in mammalian cells, COS-7 and HepG2 cells were transduced using purified baculovirus BDES particles. Blossom-like PfCSP expression was detected on both COS-7 and HepG2 cells exposed to CAG-full, CAG-205 and CMV-full, and the transduction efficacies of CAG-full and CAG-205 were clearly superior to that of CMV-full (Fig. 1C); this result might be due to codon-optimization of CAG-full and CAG-205. No signal was detected from the cells transfected with AcNPV-WT (Fig. 1C).

### Induction of anti-PfCSP Ab responses in mice using BDES-PfCSP

**Figure 2:**
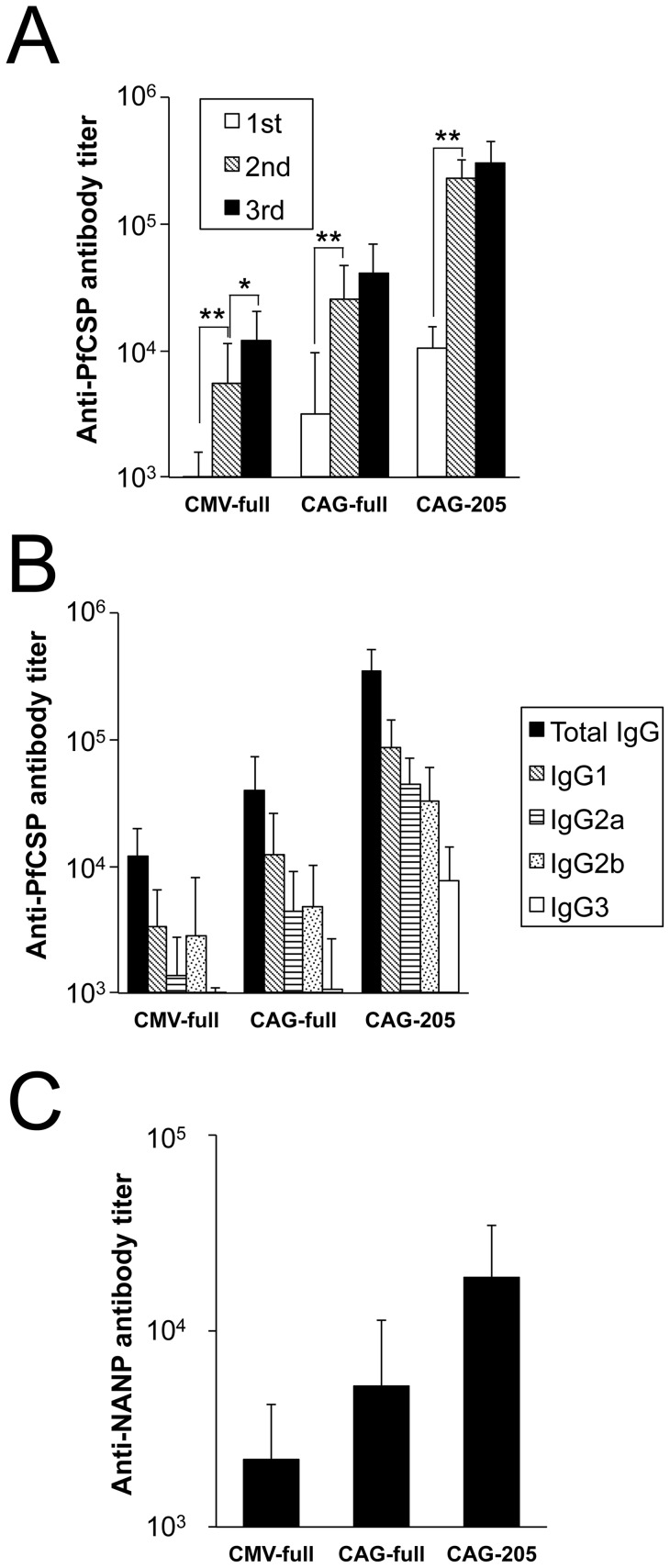
Kinetics of anti-PfCSP humoral immune responses. Groups of mice were injected with PBS or immunized intramuscularly with CMV-full, CAG-full or CAG-205 (n = 10). (*A*) Sera were collected from the immunized mice 3 weeks after the first and second immunizations and 2 weeks after the last immunization. Bars and error bars indicate the mean and SD of the values, respectively. Significant differences in each immunization group were evaluated using Student's *t*-tests. * *p*<0.05; ** *p*<0.01. (*B*) Individual sera after the last immunization were tested for total IgG, IgG1, IgG2a, IgG2b and IgG3 specific to PfCSP by ELISA. (*C*) Ab titers against the PfCSP NANP repeats in the sera after the last immunization. Statistically significant differences for each Ab titer among the three immunized groups were evaluated using the Kruskal-Wallis non-parametric tests (*p*<0.001). Data are representative of 3 independent experiments.

Three separate immunizations with BDES-PfCSP increased the anti-PfCSP total IgG titers in immunized mice (Fig. 2A). After the second booster immunization, the Abs titer against PfCSP significantly increased compared with that observed after the initial immunization, resulting in a 7.1- to 21.7-fold rise in the mean titer values (Fig. 2A). The booster effects of the third immunization were lower, but increased by 1.3- to 4.4-fold (Fig. 2A). In agreement with the amounts of PfCSP expressed on the BDES particles, the mean Ab titers ± standard deviations (SD) after the third immunization with CMV-full, CAG-full and CAG-205 were 12,053±8370, 40,433±28,480, and 303,333±139,215, respectively (Fig. 2A). Antibody subclass analysis showed that BDES-PfCSP induced not only IgG1 but also IgG2a and IgG2b (Fig. 2B), indicating that BDES induced a mixture of Th1/Th2 immune responses.

The Ab titers specific to the NANP repeat region, which are known to be associated with protection from *P. falciparum* infection [20], were induced in the sera from mice immunized with CAG-205 (18,720±15,693); this value is higher than that obtained for CAG-full (5,287±6,006) and CMV-full (2,193±1,992).

### Analysis of T cell responses against baculovirus in BDES-immunized mice

**Figure 3:**
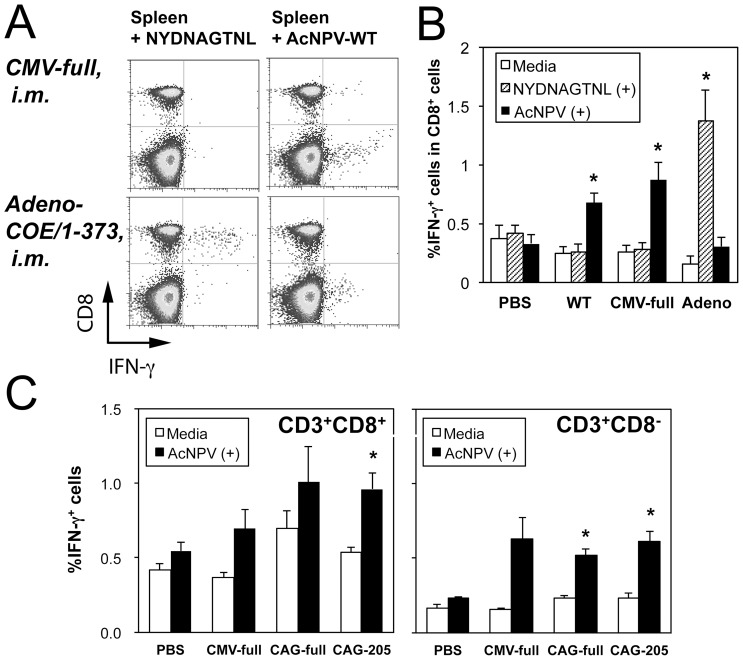
Intracellular cytokine staining of splenocytes from BDES-immunized mice. Groups of mice were injected with PBS or immunized intramuscularly (*i.m.*) with CMV-full, AcNPV-WT or Adeno-COE/1–373 (A) (B) (n = 6, experiment 5 in Table S1), or CAG-full and CAG-205 (C) (n = 3, experiment 6 in Table S1). Two weeks after the last immunization, splenocytes from each group were stimulated with either NYDNAGTNL peptides or AcNPV-WT at a MOI of 1 in duplicate wells of the cultures. After 24 hours, the incubated cells were stained with Abs against CD3 and CD8, and then stained intracellularly with anti-IFN-γ or its isotype control. (A) Representative dot plots are shown. (B) (C) Bars and error bars show the mean values and standard errors for the percentage of IFN-γ positive cells, respectively. Significant differences between non-stimulated media controls and stimulated wells were evaluated using Student's *t*-tests. * *p*<0.05.

To investigate T cell responses specific to baculovirus itself, as well as to PfCSP, splenocytes from the immunized mice were stimulated with AcNPV-WT and the H-2K^d^-restricted PfCSP peptide, NYDNAGTNL (PfCSP^39–47^) [21]. As a positive control for T cell-responses against PfCSP, the Adeno-COE/1-373, which expresses PfCSP^1–373^, was tested. The Adeno-COE/1-373 viral vector significantly induced IFN-γ^+^ cells (1.21% out of the CD8^+^ T cell population), when the splenocytes were stimulated with the CD8^+^ T cell epitope (Fig. 3A, B). BDES immunization did not induce any CS-specific T cell responses (Fig. 3A, B), whereas baculovirus-specific responses in CD3^+^CD8^+^ and CD3^+^CD8^−^ T cells were detected in mice immunized with BDES-PfCSP (Fig. 3B, C), indicating the immunogenicity of this baculovirus as a vaccine vector.

### Protection against challenge infections by the bites of mosquitoes infected with PfCSP-Tc/Pb

**Figure 4:**
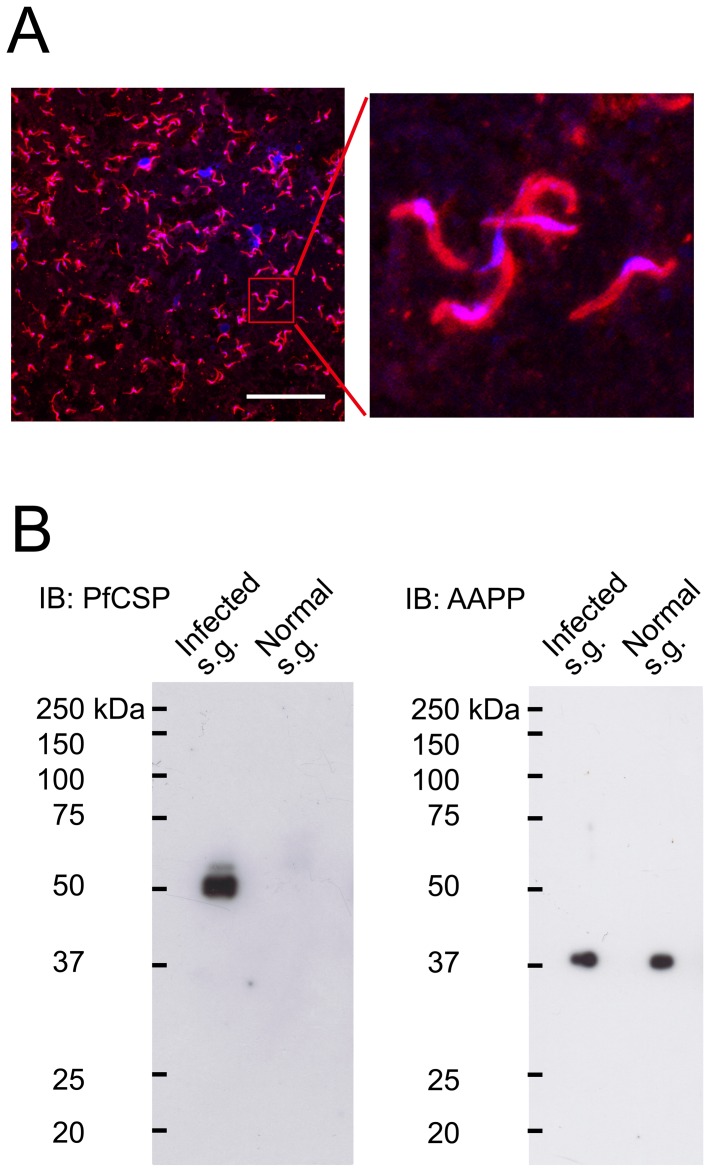
PfCSP expression in transgenic *P. berghei*. (*A*) Confocal laser scanning microscopy PfCSP expression in transgenic *P. berghei*. Salivary glands from the infected mosquitoes as well as transgenic *P. berghei* sporozoites were probed with the 2A10 mAb conjugated to Alexa Flour 594 (red). Parasite nuclei and mosquito salivary glands were visualized by DAPI (blue). Bar  = 50 µm. (*B*) Salivary glands from uninfected mosquitoes or mosquitoes infected with the transgenic parasites were lysed and loaded onto 10% gels, and then immunoblotted with the 2A10 mAb and anti-AAPP mAb (28B8).

To evaluate the vaccine efficacy of BDES-PfCSP in a murine model, a transgenic *P. berghei*, in which the full-length PbCSP was replaced with PfCSP-Tcell (PfCSP-Tc/Pb), was generated (Fig. S1). A mAb, 2A10, which recognizes the NANP repeat sequence of PfCSP, reacted strongly with the surface of the PfCSP-Tc/Pb transgenic sporozoites (Fig. 4A). Western blotting showed that parasites in the mosquito salivary glands expressed PfCSP as a triplet of bands with relative molecular masses of around 50 kDa (Fig. 4B), which may correspond to precursor and mature forms of the protein, as previously reported [22]. We also confirmed that non-infected salivary glands did not react with the 2A10 mAb, but the mosquito saliva protein AAPP, which was used as an internal control, was expressed in infected and non-infected salivary glands (Fig. 4B) [23].

**Figure 5:**
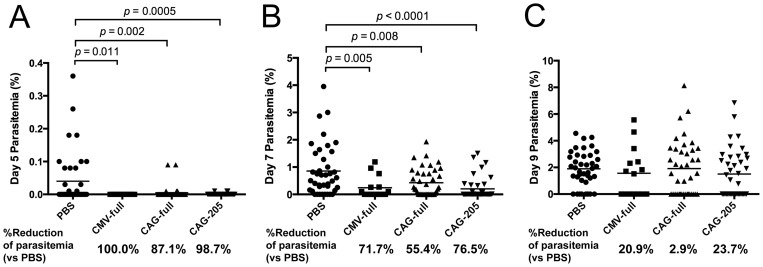
Parasitemia in the immunized mice after parasite challenge. Giemsa-stained thin smears of tail blood were prepared at days 5, 7 and 9 after challenge. The percentage parasitemias and parasitemia reduction rates in the individual mice were compared with those from the PBS-control mice using the following formula: % reduction  =  [1–(% parasitemia of immune group)/(% parasitemia of PBS group]). Data from 3 independent experiments were pooled (n = 40), except for the CMV-full group (n = 15).

Two weeks after the third immunization with BDES vaccines, the mice were challenged by the bites of PfCSP-Tc/Pb-infected mosquitoes. Malaria is transmitted by the bite of an infected mosquito and this method of transmission has been used in challenge infections in human clinical trials [24]–[27]; this method of transmission was used in the present study. BDES-PfCSP was compared with the non-immunized group for its protective efficacy. In some experiments, AcNPV-Dual-PbCSP (a PbCSP vaccine that provided 100% protection against wild-type *P. berghei* [13]), AcNPV-WT (wild-type baculovirus control for vector-related responses), or Adeno-COE/1-373 were used as immunization controls (Table S1). Significant protective efficacy was observed in the mice immunized with CMV-full (20.0%), CAG-full (26.2%) and CAG-205 (30.4%), but AcNPV-Dual-PbCSP did not confer any protection, indicating that the protective efficacy was antigen-specific (Table 1). In addition, the Adeno-COE/1–373 vaccine induced strong CD8^+^ T cell responses (Fig. 3A, B), but none of the immunized mice were protected. As observed by ourselves and other groups previously, not all of the mice in the non-immunized groups were infected because of the large variation in the numbers of sporozoites injected by individual mosquitoes [13], [28], [29]. Noticeably, five days after the challenge infections, almost all (>90%) of the mice immunized with BDES-PfCSP were uninfected, whereas 60% to 70% of those in the control groups were infected (Table 1). The vaccine delayed parasitemia could result in a reduction of parasite loads during both the liver and erythrocytic stages of the parasites. As hypothesized, BDES-PfCSP vaccines provided a significant reduction of the parasitemia 5 to 7 days after the challenge (Fig. 5A, B), but this reduction had abated at day 9 after the challenge (Fig. 5C). Notably, reduction of the parasitemia in the BDES-PfCSP immunized mice ranged from 87.1% to 100% at day 5 and 55.4% to 76.5% at day 7 as compared with that of the PBS control group, indicating that immunization with BDES-PfCSP led to delays of PfCSP-Tc/Pb-infection during the early stages of infection (Fig. 5A, B). If the parasitemias of the non-infected mice (0%) were removed from the calculation, only the day 7 parasitemia of the CAG-205 group was significantly reduced as compared with the control group, but those of the other groups were not (data not shown). The higher Ab titers against PfCSP slightly reduced the day 7 parasitemia (Fig. S2A), and there was no threshold level of anti-PfCSP titer required for complete protection (Fig. S2B, C). Similar to the anti-PfCSP titer, anti-NANP Abs were not correlated with the protective efficacy of BDES-PfCSP (Fig. S2D–F). Taken together, although the BDES vaccines delayed the appearance of blood stage parasitemia, the protective efficacy of the vaccines had no clear correlation with the anti-PfCSP Ab titers or with the anti-NANP titers.

**Table 1:** Vaccine protection levels in mice immunized with different BDES constructs following challenge infections administered by the bites of mosquitoes infected with PfCSP-Tc/Pb.

Vaccine	Delayed parasitemia: no. of uninfected mice/total no. (%)^a^			Complete protection: no. of protected mice/total no. (%)^b^	% of mice protected^e^
	5 days	7 days	9 days		
PBS	26/40 (65)	8/40 (20)	7/40 (18)	7/40 (18)^c^	0.0
AcNPV-WT	9/15 (60)	1/15 (7)	1/15 (7)	1/15 (7)	0.0
AcNPV-Dual-PbCSP	7/10 (70)	0/10 (0)	0/10 (0)	0/10 (0)	−11.1
Adeno-COE/1-373	8/10 (80)	2/10 (20)	0/10 (0)	0/10 (0)	−11.1
CMV-full	15/15 (100)	8/15 (53)	7/15 (47)	7/15 (47)^d^	20.0
CAG-full	36/40 (90)	21/40 (53)	16/40 (40)	16/40 (40)c, d	26.2
CAG-205	38/40 (95)	25/40 (63)	19/40 (48)	19/40 (48)c, d	30.4

a Mice were screened for PfCSP-Tc/Pb blood-stage infections by microscopic examination of Giemsa-stained thin smears of tail blood prepared at days 5, 7, 9 and 14 after challenge infections from PfCSP-Tc/Pb-infected mosquitoes (3≤ mosquitoes ≤7/mouse). After the appearance of parasites in the blood, all of the mice died.

b Complete protection is defined as the complete absence of blood-stage parasitemia on day 14 post- challenge.

c Cumulative data from three independent experiments (Exp. 1, 2 and 3 in Table S1).

d Each group of immunized mice were compared with the non-immunized group (PBS) to test for statistically significant differences using Fisher's exact probability test.

e Vaccine efficacy was calculated using the formula: Efficacy  =  [1–[(number of infected animals (I)vaccine/total number of animals (n)vaccine)÷(number of infected animals control (I)/total number of animals (n) control)]]*100. Mean efficacies from three independent studies are shown.

### Sporozoite-neutralizing efficacy of sera from rhesus monkeys immunized with BDES-PfCSP

In this pre-clinical evaluation, it was of great importance to investigate the type of immune responses induced by BDES in a primate model. To this end, malaria naive rhesus monkeys were immunized with CAG-full and CAG-205 vaccines as well as AcNPV-WT (Table S1). As shown in Table 2, sera from the monkeys immunized intramuscularly (*i.m*), subcutaneously (*s.c.*) or intradermally (*i.d.*) with CAG-205 exhibited marked increases in the levels of anti-PfCSP Abs (63,333, 67,000 and 81,000, respectively) compared with the controls. In contrast, the PfCSP-specific Ab titer for the CAG-full-immunization was 28,833, which is less than that obtained for the CAG-205-immunizations. In all cases, anti-NANP Ab titers were also induced by the BDES-immunization, but the titers were much lower than for anti-PfCSP. The sporozoite invasion inhibition levels were 81.1% by *s.c.* immunization with CAG-205, 98.4% by *i.m.* immunization with CAG-205, 98.4% with CAG-205 by *i.d.* and 99.9% with CAG-full by *i.m.*; in most cases these values were higher than that of the purified 2A10 mAb (97.9%). Consistent with the murine model, CAG-full-immunization of the monkeys induced low Abs titers; however, sporozoite invasion was effectively inhibited and was as high as that achieved with the CAG-205-immunizations. The present data show that all immunization routes induced high Abs responses, and that *i.m.* and *i.d.* routes were particularly effective at inhibiting sporozoite invasion.

**Table 2:** *In vitro* antibody analysis of sera from rhesus monkeys immunized with BDES-PfCSP.

Vaccine (route)	Anti-PfCSP Ab titer^a^	Anti-NANP Ab titer^a^	% Invasion efficacy b (% Inhibition)
PBS	0	0	100.0±18.1 (0)
WT (*i.m.*)	0	0	104.7±23.7 (0)
CAG-205 (*s.c.*)	63,333±42,169	4,900±2,128	18.9±4.72 (81.1)^*^
CAG-205 (*i.m.*)	67,000±33,154	6,967±5,218	1.57±0.03 (98.4)^*^
CAG-205 (*i.d.*)	81,000±32,044	7,683±4,583	1.63±0.68 (98.4)^*^
CAG-full (*i.m.*)	28,833±35,034	2,792±2,745	0.15±0.11 (99.9)^*^
2A10 mAb b	–	–	2.14±1.37 (97.9)^*^

a The anti-PfCSP Ab and anti-NANP titers of sera obtained from immunized monkeys were evaluated by ELISA. Means ± SD are shown (n = 6).

b Viable PfCSP-Tc/Pb sporozoites (5×10^3^) were incubated with pooled serum from BDES-PfCSP-immunized monkeys for 40 min on ice, and then added to cultured HepG2 cells. *P. berghei* 18S rRNA was measured by qRT-PCR 72 h after incubation. Data represent the means ± SD of the % sporozoite invasion efficacy values from cultures performed in triplicate for each group. Controls included sporozoites incubated with 25 mg/ml 2A10 mAb. Compared with the non-immune serum, all samples significantly reduced the number of the 18 S rRNA copies (*, *p*<0.01). *i.m.*: intramuscular, *s.c.*: subcutaneous, *i.d.*: intradermal.

## Discussion

In this study, we have shown that PfCSP-based baculovirus vaccines induced protective immunity against PfCSP-Tc/Pb transgenic parasites in mice, and that the antibodies induced in primates markedly inhibited sporozoite invasion of HepG2 cells. Enhancement of the PfCSP gene by codon-optimization to facilitate expression in mammalian cells clearly enhanced its expression levels in viral particles and transduced cells, leading to strong induction of humoral immunity. A principal aim when developing CSP-based vaccines is the induction of high levels of CSP Abs in the host [2], [30]. The present study, however, found that although the anti-PfCSP Ab titer in the CAG-205-immunized mice was over 8-fold higher than that of the CAG-full-immunized mice, the protection level for CAG-205 (30.4%) was not significantly different from that of CAG-full (26.2%). Many studies have reported that Abs against the NANP-repeat sequence in PfCSP can neutralize sporozoites and reduce their infectivity [20], [30]-[32]. Because anti-NANP Ab titers in the immunized groups were over 10-fold lower than the anti-PfCSP titers, most anti-PfCSP Abs induced by BDES appear to afford less protection against the parasites. Conversely, the CAG-full construct provided significant protection despite lower Ab titers, indicating the importance of the N-terminal region of PfCSP in protective immunity. Past studies on sporozoite vaccines have revealed a poor correlation between the protective efficacy of a vaccine and antibody titers. For examples, the complement fragment C3d-based PbCSP vaccine was capable of inducing strong antibody responses against CSP, but its protective efficacy, compared with the parent PbCSP vaccine, was reduced [33]. Studies in our laboratory are in progress towards development of an evaluation system which can detect the specific antibodies that contributed to the protective efficacy of a vaccine.

CD8^+^ T cells are thought to be the critical determinants of irradiated sporozoite immunity in both mice and primates [34]–[36]. The available evidences of an absence of substantial PfCSP-specific CD8^+^ T cell responses in RTS,S clinical trials strongly supports anti-PfCSP antibodies as playing a more important role in RTS,S-mediated protection than cell-mediated immunity [2]. Since BDES is designed to function not only as a VLP but also as a DNA vaccine induction of both humoral and cellular immune responses are expected. Indeed, our previous study showed that AcNPV-Dual-PbCSP could induce not only high anti-PbCSP Ab titers but also a high frequency of IFN-γ-producing T cells specific for PbCSP [13]. As BDES-PfCSP provided a mixture of Th1/Th2 immune responses (Fig. 2B), the type of immune responses induced by BDES were clearly different from that of protein-based component vaccines [15], indicating that BDES can induce both humoral and cellular immunity. In the present study, however, we could not detect anti-PfCSP specific T cell-responses in the BDES-immunized mice. In addition, the CAG-205 vaccine, which induced the strongest anti-PfCSP Ab titers and protective efficacy, lacked the N-terminal sequence comprising the H-2K^d^-restricted T cell epitope [21]. Two studies have shown that adenoviral vectors expressing PfCSP can induce CSP epitope-specific IFN-γ^+^ T cells, but the involvement in protective efficacy of such T cells remains unclear [37], [38]. In the present study, immunization with the Adeno-COE/1-373 adenoviral vaccine induced high IFN-γ^+^CD8^+^ T cell responses via stimulation by the PfCSP epitope (Fig. 3A, B), but none of the immunized mice were protected (Table 1). Consistent with our data, the Phase 1/2a clinical trial of the adenovirus-5-based vaccine encoding PfCSP reported that even though robust PfCSP-specific CD8^+^ T cell responses were induced, the vaccine did not sterilely protect volunteers against challenge via the bites of infected mosquitoes, which is the same challenge route as was used here [39]. To determine whether PfCSP epitope-specific T cells induce protective immunity in a mouse malaria model, further study is necessary to identify the role of the cytotoxic T cells during infection with PfCSP-Tc/Pb; this could be approached by using epitope-specific immunization models such as the CS epitope-pulsed dendritic cell injection model.

The present study demonstrated that BDES vaccines could induce strong anti-PfCSP Ab titers (in a primate immunization model) and that such antibodies blocked sporozoite invasion of HepG2 cells (Table 2). For BDES to be used as a vaccine in clinical settings it is important to assess its biosafety in a primate model of malarial disease. Rhesus monkeys have been used as very useful predictors of vaccine safety, immunogenicity and protective efficacy in humans as previously demonstrated [40]. After the immunization with BDES in rhesus monkeys, most animals showed transient skin erythema, skin swelling, and muscle induration from vaccination, and, in all cases, those clinical symptoms disappeared within 14 days of each injection (data not shown). Overall, no severe hematologic or biochemical reactions were observed. Previously, Jin *et al.* constructed a recombinant *Bombyx mori* baculovirus, Bmgp64HA, for expression of the HA protein of the H5N1 influenza vaccine [41]. In agreement with our findings, the study in rhesus monkeys showed that Bmgp64HA induced protection via neutralizing antibodies, which protected against influenza infection, and that the vaccine did not cause any toxicity in monkeys or mice [41]. The present large-scale study using primate and mouse models indicates that BDES vaccines could provide a sufficient quantity and quality of protective antibodies against *P. falciparum* infection. Taken together, our data show that BDES-PfCSP is safe and well tolerated with acceptable reactogenicity and systemic toxicity, thereby outlining the potential utility of this novel baculovirus dual expression system for clinical application as a malaria vaccine.

In summary, BDES-PfCSP provided strong humoral immunity against PfCSP and significant protection against PfCSP-Tc/Pb in a murine malaria model, whereas our studies with rhesus monkeys indicated that BDES-PfCSP provided high levels of anti-PfCSP Abs with strong inhibitory activity against sporozoite invasion of HepG2 cells. Compared with the current protein-based subunit and viral-vectored vaccines, BDES has several advantages, for example: BDES (i) can display full length PfCSP multimer complex on the surface of viral virions whilst maintaining its native 3D conformation; (ii) can activate innate immunity in dendritic cells through Toll-like receptor 9-dependent and -independent pathways [10]; (iii) is a safe non-replicating vector; (iv) has no-preexisting Abs against it, and (v) is cost-effective and easy to manipulate. To advance BDES to clinical trials, the 48% protection level in the murine model described in this study needs to be improved. Studies are now in progress to develop multistage and multivalent BDES malaria vaccines with the aim of improving their efficacy through use of genetically modified baculoviruses.

## Supporting Information

Figure S1 Generation of the PfCSP-Tc/Pb transgenic parasite line. Schematic representation of pBS-5′ UTR-PfCSP-Tcell-DHFR-3′ UTR, the DNA construct used for integration into the *P. berghei* genome. This construct disrupts the *pbcsp* gene and introduces the *pfcsp-tcell* gene under the control of the 5′ and 3′ UTR of the *pbcsp* gene. The signal peptide sequence of PfCSP-Tcell (A361E) was replaced with that of PbCSP.
(TIF)

Figure S2 Relationship between PfCSP-specific Ab titer and protective immunity. (A) Correlation between anti-PfCSP Ab titer and parasitemia at day 7 in mice immunized with CMV-full, CAG-full and CAG-205. (Spearman's rank correlation; *r* = −0.155; *p* = 0.133) (B) The Ab titers against PfCSP in the sera from either infected mice (black) or protected mice (white) were shown. (C) Mean Ab titers ± S.D. of (B). (D) Correlation between anti-NANP Ab titer and parasitemia at day 7 in mice immunized with CMV-full, CAG-full and CAG-205 (Spearman's rank correlation; *r* = 0.070; *p* = 0.498). (E) The Ab titers against NANP in the sera from the infected mice (black) or protected mice (white) are shown. (F) Mean Ab titers ± S.D. of (E). Data from 3 independent experiments were pooled (n = 40 for CAG-full and CAG-205, and n = 15 for CMV-full).
(TIF)

Figure S3 ELISA conditions used in this study. (A) ELISA plates were coated with either rPfCSP or NANP peptides. The anti-PfCSP mAb 2A10 was serially diluted and applied to the wells. Mean values ± S.D. from quadruplicate wells are shown. (B) Mean ± S.D. of the 2A10 concentration at the end point (OD_414_ = 0.15) at (A). (C) Serum from a naïve mouse was collected and examined by ELISA using the rPfCSP and the NANP repeat. (D) Mean ± S.D. of end point dilution at (C).
(TIF)

Materials and Methods S1 (DOC)

Table S1 Immunization groups of animals with BDES vaccines.
(DOC)

Table S2 Condition of the PfCSP-Tc/Pb infection in mice.C.
(DOC)
