# Supplementary figures and images for: Protective Efficacy of Baculovirus Dual Expression System Vaccine Expressing Plasmodium falciparum Circumsporozoite Protein
Source: PLoS One. 2013 Aug 12;8(8):e70819. doi: 10.1371/journal.pone.0070819 (PMC3741388; doi:10.1371/journal.pone.0070819)

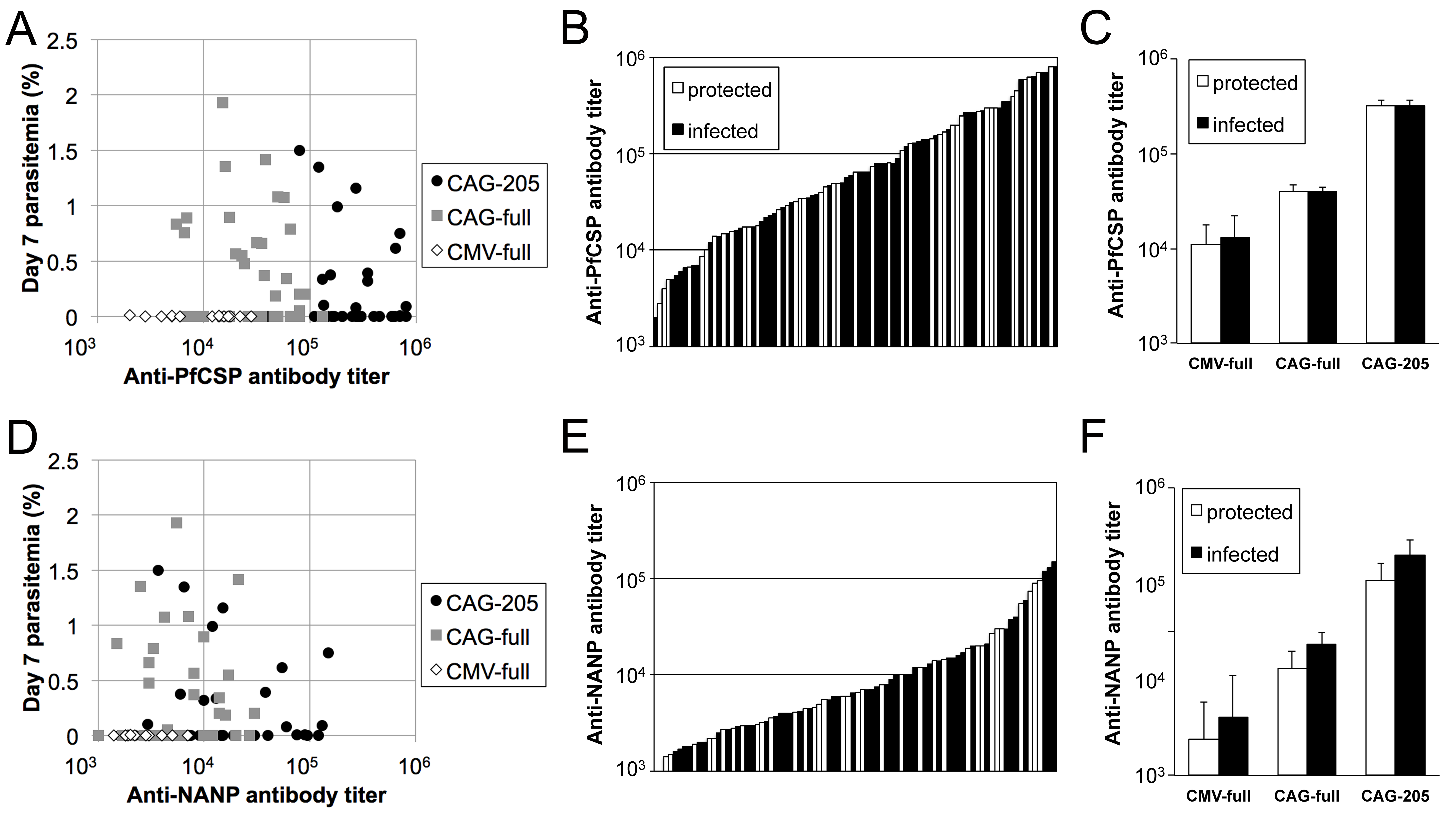

Supplement: Figure S2 — Relationship between PfCSP-specific Ab titer and protective immunity. (A) Correlation between anti-PfCSP Ab titer and parasitemia at day 7 in mice immunized with CMV-full, CAG-full and CAG-205. (Spearman's rank correlation; r = −0.155; p = 0.133) (B) The Ab titers against PfCSP in the sera from either infected mice (black) or protected mice (white) were shown. (C) Mean Ab titers ± S.D. of (B). (D) Correlation between anti-NANP Ab titer and parasitemia at day 7 in mice immunized with CMV-full, CAG-full and CAG-205 (Spearman's rank correlation; r = 0.070; p = 0.498). (E) The Ab titers against NANP in the sera from the infected mice (black) or protected mice (white) are shown. (F) Mean Ab titers ± S.D. of (E). Data from 3 independent experiments were pooled (n = 40 for CAG-full and CAG-205, and n = 15 for CMV-full). (TIF) [file pone.0070819.s002.tif]

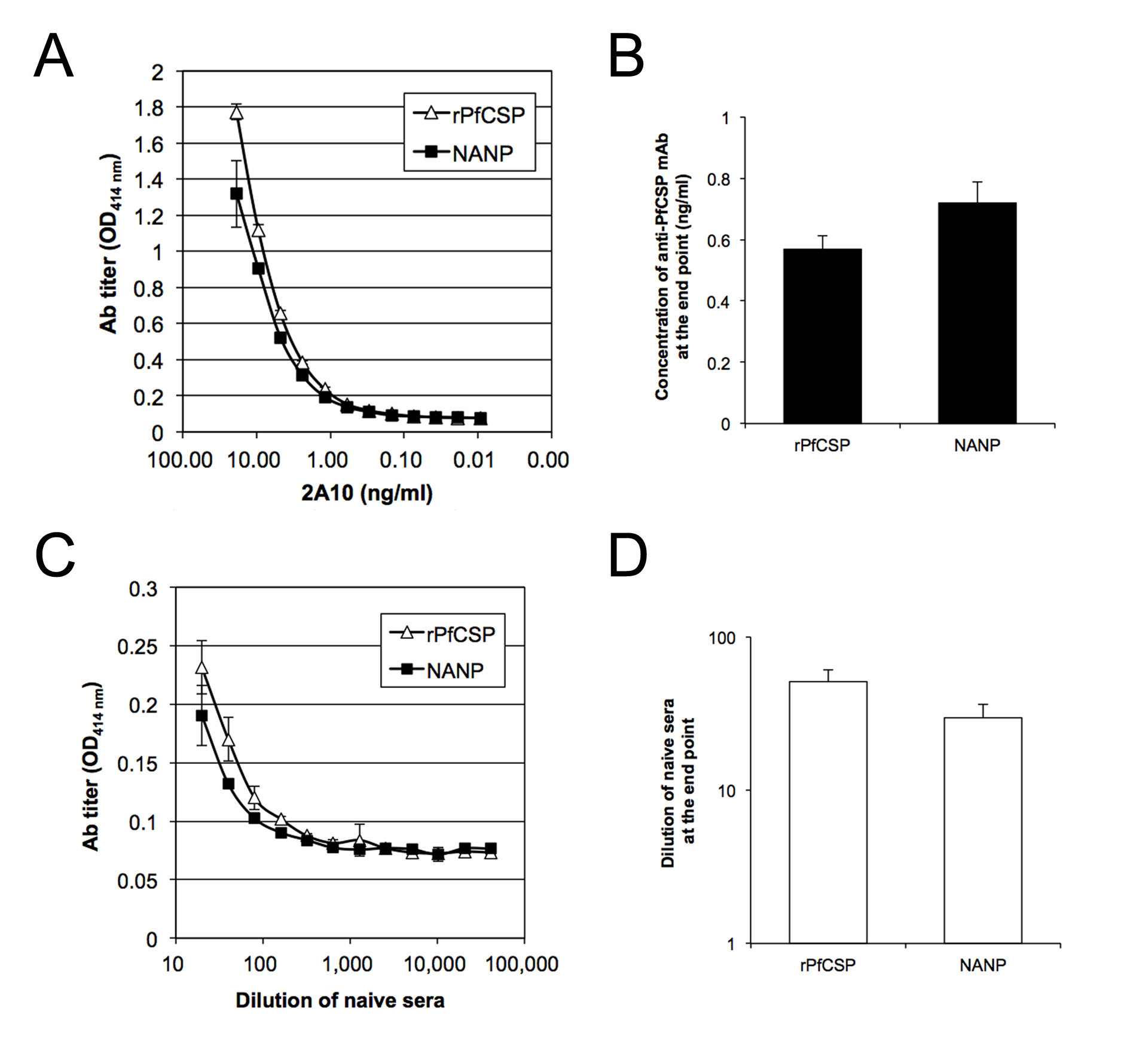

Supplement: Figure S3 — ELISA conditions used in this study. (A) ELISA plates were coated with either rPfCSP or NANP peptides. The anti-PfCSP mAb 2A10 was serially diluted and applied to the wells. Mean values ± S.D. from quadruplicate wells are shown. (B) Mean ± S.D. of the 2A10 concentration at the end point (OD414 = 0.15) at (A). (C) Serum from a naïve mouse was collected and examined by ELISA using the rPfCSP and the NANP repeat. (D) Mean ± S.D. of end point dilution at (C). (TIF) [file pone.0070819.s003.tif]
