# Supplementary material for: Protective Efficacy of Baculovirus Dual Expression System Vaccine Expressing Plasmodium falciparum Circumsporozoite Protein
Source: PLoS One. 2013 Aug 12;8(8):e70819. doi: 10.1371/journal.pone.0070819 (PMC3741388; doi:10.1371/journal.pone.0070819)
Supplement: Materials and Methods S1 — (DOC) [file pone.0070819.s004.doc]

**Material and Methods S1.**

***Animals, cell lines, parasites, mosquitoes and antibodies (Ab)***

Female BALB/c mice were obtained from Japan SLC Inc. (Hamamatsu, Japan) and used at 7 weeks of age in all of the experiments. Healthy Rhesus monkeys (5-10 years of age) were provided by the Armed Forces Research Institute of Medical Sciences (AFRIMS) Department of Veterinary Medicine. The study using Rhesus monkeys was conducted at AFRIMS, Bangkok, Thailand.

Sf9, COS7 and HepG2 cells were maintained as previously described . Transgenic PfCSP-Tcell (Tc)/Pb parasites were generated to replace the PbCSP gene with PfCSP^A361E^ using the same methods as described previously (Fig. S1, ). *Anopheles stephensi* mosquitoes (SDA 500 strain) were maintained as described previously .

mAb 2A10, which recognizes the central NANP repeat sequence of PfCSP was obtained from MR4 (Manassas, VA). An anti-VP39 polyclonal Ab was generated from rabbits immunized with a synthetic peptide corresponding to the amino acid sequence of VP39^36-48^ (CSPDAYHDDGWFI, custom made, Sigma-Aldrich, St Louis, MO).

***Immunoblotting***

BDES-PfCSP cell lysates as well as the salivary glands from parasite-infected mosquitoes were separated by 8% SDS-PAGE and transferred to polyvinylidene fluoride membranes, and then probed with the anti-VP39 Ab or anti-AAPP mAb (28B8). Secondary Abs were used to detect primary Ab binding as described previously . The membrane was stripped, and then reblotted with the 2A10 mAb.

***Confocal laser scanning microscopy***

COS-7 (10^4^ cells/well) and HepG2 cells (4 × 10^4^ cells/well) were transduced with purified baculoviruses at multiplicity of infections (MOIs) of 500 and 100, respectively. After 48 hours incubation with the viruses, the cells were incubated with Alexa Fluor 594-conjugated 2A10 mAb and Syto-13 nucleic acid dye (Invitrogen). Dissected salivary glands were fixed on glass slides using a solution of cold acetone/methanol (4:6). The slides were stained with the 2A10 mAb, and mounted with a drop of Vectashield containing 4’,6’-diamidino-2-phenylindole (DAPI). An LSM710 inverted laser scanning microscope (Carl Zeiss, Tokyo, Japan) using 20× and 40× objectives was used for image acquisition.

***Enzyme-linked immunosorbent assay (ELISA)***

For the production of recombinant PfCSP protein, *pfcsp* cDNA was subcloned into pET32-b (+) (Invitrogen) and expressed as a thioredoxin fusion protein. rPfCSP protein was purified by affinity chromatography on a Ni-NTA column (Qiagen, Valencia, CA) followed by dialysis against PBS as described previously . Sera from immunized mice were collected from tail blood samples 3 weeks after the first and second immunizations and 2 weeks after the third. PfCSP-specific Abs levels were quantified by ELISA. Precoated EIA/RIA plates (Corning, Corning, NY) with 0.4 µg/well of rPfCSP or 0.1 µg/well of NANP peptides [(NANP)_4_NDPC, custom made, Sigma] were blocked with 1% BSA in PBS and incubated with serial dilutions of sera from the immunized and control mice. Total IgG, IgG1, IgG2a, IgG2b and IgG3 specific for the above antigens were detected using HRP-conjugated anti-mouse IgGs as described previously . Endpoint titers were expressed as the reciprocal of the last dilution, which gave an optical density at 414 nm of 0.15 U above the values of the negative controls (< 0.1). The ELISA conditions used in this study are shown in Fig. S3. All mice used were seronegative prior to immunization.

Ab responses against the rPfCSP and NANP peptides in the Rhesus monkeys were assessed by ELISA using HRP-conjugated anti-monkey IgGs (A-2054, Sigma).

***Sporozoite neutralization assays***

HepG2 cells were seeded at a density of 5 × 10^4^ cells per well in a collagen type I-coated eight-well chamber slide (BD Biosciences, San Jose, CA) 48 h prior to addition of the sporozoites. The sporozoite neutralization assay was performed using previously described methods . Sporozoites (5 × 10^3^ cells) isolated from mosquito salivary glands were incubated with the pooled sera from the monkeys, after which they were added to the HepG2 cultures. After 72 h incubation, total RNA was extracted using a QIAamp RNA blood mini kit (Qiagen, Hiden, Germany), and quantification of *P. berghei* 18S rRNA was determined using real time reverse transcription-PCR as described previously .

***Intracellular cytokine staining***

Splenocytes were obtained from mice immunized intramuscularly with BDES vaccines or Adeno-COE/1-373 two weeks after the last immunization. After the red blood cells were lysed, the splenocytes were stimulated for 24 hours with the H-2K^d^-restricted PfCSP peptide NYDNAGTNL (PfCSP^39-47^) or AcNPV-WT at a MOI of 1 in RPMI 1640 medium supplemented with 10% FBS, 100 U/ml penicillin, 100 µg/ml streptomycin, 1 mM pyruvate, 10 mM HEPES, 0.1 mM nonessential amino acids, and 50 µM 2-ME. Before staining, the cells were blocked with anti-mouse CD16/32 and mouse IgG. The cells were stained with Alexa Fluor 647-conjugated anti-CD3ε (145-2C11; Biolegend, San Diego, CA) and PerCP/Cy5.5-conjugated anti-CD8β (YTS156.7.7; Biolegend). The cells were fixed and permeabilized using a Cytofix/cytoperm kit (BD Biosciences). After washing, cells treated in this manner were incubated with FITC-conjugated anti-IFN-γ (XMG1.2; Biolegend) or a FITC-conjugated Rat IgG1 isotype control (RTK2071, Biolegend). FACSVerse (BD Biosciences) and FlowJo software (Tree Star, Ashland, OR) were used to analyze the cells.

**References**

1. Yoshida S, Nagumo H, Yokomine T, Araki H, Suzuki A, Matsuoka H. Plasmodium berghei Circumvents Immune Responses Induced by Merozoite Surface Protein 1- and Apical Membrane Antigen 1-Based Vaccines. PLoS One **2010**; 5:e13727.

2. Yoshida S, Kawasaki M, Hariguchi N, Hirota K, Matsumoto M. A baculovirus dual expression system-based malaria vaccine induces strong protection against Plasmodium berghei sporozoite challenge in mice. Infect Immun **2009**; 77:1782-9.

3. Sumitani M, Kasashima K, Yamamoto D, et al. Reduction of malaria transmission by transgenic mosquitoes expressing an anti-sporozoite Ab in their salivary glands. Insect Molecular Biology **2012**; in press.
