# Supplementary material for: Protective Efficacy of Baculovirus Dual Expression System Vaccine Expressing Plasmodium falciparum Circumsporozoite Protein
Source: PLoS One. 2013 Aug 12;8(8):e70819. doi: 10.1371/journal.pone.0070819 (PMC3741388; doi:10.1371/journal.pone.0070819)
Supplement: Table S1 — Immunization groups of animals with BDES vaccines. (DOC) [file pone.0070819.s005.doc]

***TABLE S1.*** *Immunization groups of animals with BDES vaccines.*

| **Expt** | **Strain** | **Group** | **Vaccine** | **Dose** | **Route^a^** | **N** | **Numbers of mosquitoes ingesting blood / mouse^b^** | **Readouts** |
| --- | --- | --- | --- | --- | --- | --- | --- | --- |
| 1 | Balb/c | 1 | PBS | - | *i.m.* | 15 | 4.5 ± 0.8 | Challenge infection study with PfCSP-Tc/Pb |
|  |  | 2 | CAG-full | 10^8^ PFU | *i.m.* | 15 | 4.6 ± 0.8 |  |
|  |  | 3 | CAG-205 | 10^8^ PFU | *i.m.* | 15 | 4.1 ± 0.9 |  |
|  |  | 4 | AcNPV-WT | 10^8^ PFU | *i.m.* | 15 | 4.9 ± 0.9 |  |
| 2 | Balb/c | 1 | PBS | - | *i.m.* | 10 | 3.7 ± 1.3 | Challenge infection study with PfCSP-Tc/Pb |
|  |  | 2 | CAG-full | 10^8^ PFU | *i.m.* | 10 | 3.9 ± 1.1 |  |
|  |  | 3 | CAG-205 | 10^8^ PFU | *i.m.* | 10 | 4.7 ± 1.3 |  |
|  |  | 4 | AcNPV-Dual-PbCSP | 10^8^ PFU | *i.m.* | 10 | 3.3 ± 0.5 |  |
|  |  | 5 | Adeno-COE/1-373 | 10^10^ VP | *i.m* | 10 | 3.4 ± 0.7 |  |
| 3 | Balb/c | 1 | PBS | - | *i.m.* | 15 | 3.5 ± 0.7 | Challenge infection study with PfCSP-Tc/Pb |
|  |  | 2 | CAG-full | 10^8^ PFU | *i.m.* | 15 | 3.8 ± 0.9 |  |
|  |  | 3 | CAG-205 | 10^8^ PFU | *i.m.* | 15 | 3.5 ± 0.6 |  |
|  |  | 4 | CMV-full | 10^8^ PFU | *i.m.* | 15 | 3.7 ± 0.7 |  |
| 4 | Rhesus monkey | 1 | PBS (5% sucrose) | - | *i.m.* | 3 | - | Immune responses |
|  |  | 2 | AcNPV-WT | 3 × 10^9^ PFU | *i.m.* | 3 | - |  |
|  |  | 3 | CAG-205 | 3 × 10^9^ PFU | *s.c.* | 6 | - |  |
|  |  | 4 | CAG-205 | 3 × 10^9^ PFU | *i.m.* | 6 | - |  |
|  |  | 5 | CAG-205 | 3 × 10^9^ PFU | *i.d.* | 6 | - |  |
|  |  | 6 | CAG-full | 3 × 10^9^ PFU | *i.m.* | 6 | - |  |
| 5 | Balb/c | 1 | PBS | - | *i.m.* | 6 | - | IFN-γ intracellular cytokine staining |
|  |  | 2 | AcNPV-WT | 10^8^ PFU | *i.m.* | 6 | - |  |
|  |  | 3 | CMV-full | 10^8^ PFU | *i.m.* | 6 | - |  |
|  |  | 4 | Adeno-COE/1-373 | 10^8^ PFU | *i.m.* | 6 | - |  |
| 6 | Balb/c | 1 | PBS | - | *i.m.* | 3 | - | IFN-γ intracellular cytokine staining |
|  |  | 2 | CMV-full | 10^8^ PFU | *i.m.* | 3 | - |  |
|  |  | 3 | CAG-full | 10^8^ PFU | *i.m.* | 3 | - |  |
|  |  | 4 | CAG-205 | 10^8^ PFU | *i.m.* | 3 | - |  |

^a^ *i.m.* = intramuscular; *s.c.* = subcutaneous; *i.d.* = intradermal

^b^ Means ± S.D. are shown.
