# Supplementary material for: Protective Efficacy of Baculovirus Dual Expression System Vaccine Expressing Plasmodium falciparum Circumsporozoite Protein
Source: PLoS One. 2013 Aug 12;8(8):e70819. doi: 10.1371/journal.pone.0070819 (PMC3741388; doi:10.1371/journal.pone.0070819)
Supplement: Table S2 — Condition of the PfCSP-Tc/Pb infection in mice.C. (DOC) [file pone.0070819.s006.doc]

**TABLE S2.**

| **Expt** | **Strain** | **Group** | **Vaccine** | **N** | **Parasitemia (%) mean ± S.E.^a^** | | | **No. of non-infected mice/total no. (%)^b^** |
| --- | --- | --- | --- | --- | --- | --- | --- | --- |
|  |  |  |  |  | **Day 5** | **Day 7** | **Day 9** |  |
| 1 | Balb/c | 1 | PBS | 15 | 0.06 ± 0.02 | 0.63 ± 0.17 | 1.84 ± 0.90 | 1/15 (6) |
| 2 | Balb/c | 1 | PBS | 10 | 0.05 ± 0.04 | 1.58 ± 0.41 | 1.88 ± 0.44 | 1/10 (10) |
| 3 | Balb/c | 1 | PBS | 15 | 0.02 ± 0.01 | 0.60 ± 0.17 | 1.96 ± 0.42 | 5/15 (33) |

^a^ Giemsa-stained thin smears of tail blood were prepared at days 5, 7 and 9 after challenge.

^b^ Numbers of non-infected mice are defined as the complete absence of blood-stage parasitemia on day 14 post-challenge.
